# Tissue Renin–Angiotensin Systems: A Unifying Hypothesis of Metabolic Disease

**DOI:** 10.3389/fendo.2014.00023

**Published:** 2014-02-28

**Authors:** Jeppe Skov, Frederik Persson, Jørgen Frøkiær, Jens Sandahl Christiansen

**Affiliations:** ^1^Department of Endocrinology and Internal Medicine, Aarhus University Hospital, Aarhus, Denmark; ^2^Novo Nordisk A/S, Bagsvaerd, Denmark; ^3^Steno Diabetes Center, Gentofte, Denmark; ^4^Department of Clinical Physiology and Molecular Imaging, Aarhus University Hospital, Aarhus, Denmark; ^5^Department of Clinical Medicine, Aarhus University, Aarhus, Denmark

**Keywords:** renin–angiotensin system, diabetes, metabolic syndrome, obesity, GLP-1, cortisol, vitamin D, osteoporosis

## Abstract

The actions of angiotensin peptides are diverse and locally acting tissue renin–angiotensin systems (RAS) are present in almost all tissues of the body. An activated RAS strongly correlates to metabolic disease (e.g., diabetes) and its complications and blockers of RAS have been demonstrated to prevent diabetes in humans. Hyperglycemia, obesity, hypertension, and cortisol are well-known risk factors of metabolic disease and all stimulate tissue RAS whereas glucagon-like peptide-1, vitamin D, and aerobic exercise are inhibitors of tissue RAS and to some extent can prevent metabolic disease. Furthermore, an activated tissue RAS deteriorates the same risk factors creating a system with several positive feedback pathways. The primary effector hormone of the RAS, angiotensin II, stimulates reactive oxygen species, induces tissue damage, and can be associated to most diabetic complications. Based on these observations, we hypothesize that an activated tissue RAS is the principle cause of metabolic syndrome and type 2 diabetes, and additionally is mediating the majority of the metabolic complications. The involvement of positive feedback pathways may create a self-reinforcing state and explain why metabolic disease initiate and progress. The hypothesis plausibly unifies the major predictors of metabolic disease and places tissue RAS regulation in the center of metabolic control.

## Introduction

The renin–angiotensin system (RAS) is classically considered to play key roles in regulating blood pressure as well as water and sodium balance. Liver secreted angiotensinogen (AGT) is enzymatically cleaved to angiotensin (ANG) I by kidney-derived renin. ANG I is, hereafter, cleaved by angiotensin converting enzyme (ACE) to the effector hormone ANG II. This classical theory has, however, been fundamentally revised in recent years. It is now evident that the components of RAS, in addition to the classical pathway, are produced and acting locally in multiple tissues; a concept known as tissue RAS. The local effects are diverse and depend on the specific tissues involved. Additional components (angiotensin peptides, receptors, and enzymes) of the system have been identified but ANG II is still believed to exert the most important actions via the ANG II receptor type 1 (AT_1_R). Although many details on tissue RAS have been elucidated, the main function of the systems still remains partly unknown. For reviews on tissue RAS see Ref. (1–3).

It is well established that an activated RAS is a major risk factor of both cardiovascular (4) and renal disease (5). Inhibitors of RAS [ACE inhibitors, ANG II receptor blockers (ARB), and renin inhibitors] are therefore widely used in the clinic. The RAS is also closely associated to the metabolic syndrome (6) and recently, inhibitors of RAS have shown to prevent the onset of type 2 diabetes (T2D) in high risk populations (7, 8).

These latter findings suggest an important role of RAS in metabolic disease. However, compared to hormones such as glucagon-like peptide-1 (GLP-1) or cortisol, the RAS may seem only a weak regulator of metabolism. We will challenge this perception and argue that the tissue RAS can be considered as the most central player in metabolic regulation.

This hypothesis originates from reported studies of the tissue RAS regulation. A range of metabolic potent hormones and conditions closely interact with tissue RAS and it is often hard to distinguish direct hormonal actions from actions secondary to tissue RAS stimulation/inhibition. Because of this consistent convergence toward tissue RAS and the well-known potent actions of especially ANG II, we hypothesize that the effects are mediated through regulations of tissue RAS. Implications of this hypothesis may not only change our view on endocrine physiology but also explain both the origin of some metabolic diseases and the accompanying complications.

## Tissue RAS Regulation

Below, we will review the interactions between tissue RAS and some of the main players in metabolic disease [hyperglycemia, obesity, hypertension, exercise, GLP-1, cortisol, and vitamin D (VitD)].

### Hyperglycemia stimulates tissue RAS and vice versa

Patients with diabetes are characterized by an impaired ability to secrete insulin and/or a decreased sensitivity to insulin. T2D and metabolic syndrome have consistently been related to an activated RAS, and several *in vitro* studies find hyperglycemia to stimulate tissue RAS in different tissues (9–14). Renin release after GPR91 receptor activation with succinate may partly be the mechanism of action (15).

A recent 26-weeks randomized controlled trial found that the ARB valsartan improves both beta cell function and insulin sensitivity in subjects with impaired glucose metabolism (16). The large NAVIGATOR study found valsartan treatment to relatively reduce the incidence of diabetes by 14% compared to placebo in patients with impaired glucose tolerance during 5 years follow up (8). The DREAM study did not find ACE inhibition with ramipril for 3 years to significantly reduce the incidence of diabetes but it did increase regression to normoglycemia (17). Several other studies in both animals and humans find that ANG II decreases insulin secretion and sensitivity while these are improved by RAS inhibitors. Conflicting results do exist, however, with regard to insulin sensitivity, as recently reviewed (18, 19).

In addition to acutely diminished insulin release, ANG II decreases beta cell proliferation and stimulates beta cell apoptosis leading to impaired long-term islet function (20).

### Obesity stimulates tissue RAS and vice versa

It is well-known that obesity predisposes to metabolic disease and that many T2D patients are obese. Several studies in humans and animals find obesity associated with enhanced activity of both systemic RAS and adipose tissue RAS (21). Especially, the AGT synthesis is very developed in adipocytes and contributes significantly to the systemic pool (22). The activity of tissue RAS is higher in visceral/central adipose tissue than in subcutaneous tissue (23), which may explain the risks related to upper body visceral fat accumulation (24).

Renin–angiotensin system components are complexly involved in the development of obesity by affections of satiety, energy expenditure, and adipocyte growth and differentiation (21). ARBs reduce body weight in both rodents and patients (25, 26). Surprisingly, chronic ANG II infusion also decreases body weight in rodents (27). However, adipose specific over-activation of AGT expression increases body weight in mice (28), which may be the setting that mimics obesity related RAS activation the best. The apparently contradicting findings probably illustrate the importance of the local nature of RAS in adipose tissue.

### Hypertension stimulates tissue RAS and vice versa

It is well-known that ANG II induces hypertension through vasoconstriction and sodium retention. Hypertension, however, also activates tissue RAS through mechanical stretch. This has been shown *in vitro* and *in vivo* in several studies in cardiac myocytes (29) and mesangial cells (30) as well as in skeletal muscle myoblasts (31). Generally, the mechanical stretch is found to up-regulate tissue RAS synthesis of AGT, ANG II, and AT_1_Rs.

### GLP-1 inhibits tissue RAS and vice versa

GLP-1 and ANG II have multiple different actions in a variety of tissues that are not included in the classical view of either of the hormones. Interestingly, when the topic is studied in detail, all GLP-1 actions seem to be counteracted by ANG II. We therefore question whether the two systems are independent or if GLP-1 actions partly or totally depend on the more widely distributed tissue RAS. This dependence is supported by several studies.

The circulatory ANG II levels decrease in response to GLP-1 infusion in healthy subjects (32). Exendin-4 (GLP-1R agonist) attenuated the effect of ANG II-induced hypertension in mice (33) and *in vitro* GLP-1 effectively inhibited ANG II-induced mesangial cell damage (34). GLP-1 and the ARB candesartan additively prevent β-cell apoptosis through the same signaling pathway (20). A dipeptidyl peptidase-4 inhibitor and valsartan additively improve both β-cell structure and function in T2D mice (35). Finally, a recent study provides a novel biochemical pathway on how GLP-1 inhibits ANG II signaling by demonstrating that GLP-1R agonists induce protective actions in glomerular endothelium cells by inhibiting the post-receptor signaling pathway of ANG II at phospho-c-Raf(Ser338) via phospho-c-Raf(Ser259) (12). Thus, it seems likely that GLP-1 from both a functional and biochemical perspective is an inhibitor of ANG II actions. Since it is difficult to identify GLP-1 actions that are not counteracted by ANG II, it is tempting to hypothesize that GLP-1 acts primarily through down-regulation of tissue RAS.

T2D patients have an impaired incretin effect (36), low levels of GLP-1 (37), and an impaired response to GLP-1 (38). In a rat model of metabolic syndrome, ARBs were found to increase fasting plasma levels of GLP-1 by 2.5-fold and increase pancreatic GLP-1R expression (39). This strongly suggests an AT_1_R-mediated decrease in GLP-1 synthesis and GLP-1R expression.

### Cortisol stimulates tissue RAS and vice versa

Glucocorticoids are steroid hormones with a wide range of important and essential actions. The hormones’ marked impact on metabolism is revealed in Cushing’s syndrome where excess cortisol induces central obesity, hypertension, osteoporosis, and diabetes. These manifestations are similar to those of increased RAS activity.

Glucocorticoids stimulate AGT transcription and secretion in adipocytes (40), kidney tubular cells (41, 42), and cardiac fibroblasts (43). In addition, glucocorticoids up-regulate AT_1_R expression in vascular smooth muscle cells but do not seem to directly affect post-receptor signaling (44, 45).

Besides ANG II’s well-known actions on adrenal aldosterone secretion, it also stimulates cortisol secretion (46, 47).

The above indicates that the metabolic effects of glucocorticoids may partly be mediated via up-regulation of tissue RAS. The anti-inflammatory properties of glucocorticoids, however, cannot be explained by tissue RAS regulation.

### Vitamin D inhibits tissue RAS and (maybe) vice versa

Vitamin D has become an important topic in metabolic research and VitD deficiency has been linked to insulin resistance and impaired beta cell function (48). The VITAL study found that VitD receptor activation reduced albuminuria in T2D patients in a fashion comparable to RAS-inhibition (49). Several human studies report an inverse relationship between VitD levels and circulating RAS activity (50, 51). In VitD receptor null mice, the renin expression and plasma ANG II concentration are increased several-fold (52). *In vitro* VitD inhibits pancreatic islet RAS component synthesis in parallel to the improvement in beta cell secretory function (53). The VitD induced suppression of renin gene transcription is believed partly to be the mechanism of action (54). Considerable additional evidence supports the theory of VitD induced inhibition of RAS at both systemic and tissue level; for reviews see (54, 55).

In an interesting recent paper, the close interactions between VitD and the RAS are reviewed from both a functional and evolutionary perspective (56). It is proposed that increased RAS activity negatively regulates the VitD level through inflammatory responses and that this explain the pandemic of VitD deficiency (56). However, the specific mechanisms underlying this effect are not documented.

### Regular exercise inhibits tissue RAS

It is well established that moderate aerobic exercise reduces the risk of both metabolic and cardiovascular disease in humans (57). Studies in rodents find chronic exercise to inhibit tissue RAS activation in the brain (58) (reduced local ACE and AT_1_R), heart (59, 60) (reduced local ACE and ANG II), and kidney (reduced local AT_1_R) (61). These findings suggest that down-regulation of tissue RAS may partly mediate the beneficial effects related to frequent exercise.

During exercise, however, tissue RAS seem to be acutely activated in the kidneys (increased ANG II, ACE, and AGT), which may function to redistribute blood flow to the muscles (62).

The mechanism behind the different effects of long-term chronic exercise and short-term acute exercise on tissue RAS activity is not fully elucidated but may be related to the differences in sympathetic tone between the two situations. The sympathetic tone increases acutely during exercise whereas regular exercise has shown to decrease an elevated sympathetic tone (63). In the kidneys, at least, RAS activity increases in response to sympathetic stimulation.

### Other hormones and tissue RAS

Angiotensin II and vasopressin are both vasoconstrictors and antidiuretics. In the kidneys, ANG II and vasopressin stimulate aquaporin-2 insertion in the inner medulla through the AT_1_R and V_2_ receptor, respectively. Interestingly, in AT_1_R knock-out mice the vasopressin-induced signaling and aquaporin-2 insertion are defective (64). This might reflect a dependence of vasopressin on the tissue RAS.

In the kidney, dopamine functions primarily through the D_1_ receptor, which is a G-protein coupled receptor similar to the GLP-1R. Like GLP-1, and in contrast to ANG II, dopamine inhibits tubular salt reabsorption and seems to prevent kidney damage (65). Interestingly, dopamine is found to inhibit AGT synthesis in proximal tubule cells (65), suppress renin expression (66), and down-regulate AT_1_Rs (67, 68).

## Hypothesis

### Tissue RAS is central to cell regulation, and the effect of multiple hormones depend on the system

Multiple hormones and conditions interact closely with the widely distributed tissue RAS. The local effects of ANG II show great diversity in different tissues and often mimic or oppose the actions of the hormones, which regulate tissue RAS.

We therefore hypothesize that the apparent actions of many hormones are induced through alterations in tissue RAS activity. Thus, it is the cellular pathways of tissue RAS that in the end effect actions. The dependence can be direct and strong (e.g., GLP-1) or partial and less direct (e.g., VitD) mediated via regulations of genes coding for RAS components.

Especially hormones acting through G-Protein coupled receptors seem to be likely candidates for direct tissue RAS dependent hormones. The nature of the receptor (RAS stimulating or inhibiting) along with the distribution will determine the effects of the hormone.

### Increased tissue RAS activity is responsible for metabolic disease

The observation of a relationship between RAS and metabolic disease is not new (6, 69). An activated RAS promotes all the characteristic properties of metabolic syndrome [hypertension, hyperglycemia, insulin resistance, obesity, and dyslipidemia (6)]. The novelty lies in the explicit coupling between tissue RAS and the major risk factors of metabolic disease. If tissue RAS completely or partly underlies the actions of GLP-1, VitD, and cortisol as hypothesized, then the metabolic power of the system is greater than first anticipated. Therefore, we propose that an activated tissue RAS is mandatory in the pathogenesis of most metabolic diseases, including T2D. The different degrees of tissue RAS activation in different tissues will determine the phenotype of the disease.

The activity of RAS also rises physiologically during pregnancy, which could partly explain the origin of gestational diabetes in predisposed individuals (70). In addition, preeclampsia is potentially the result of extreme RAS activation (70).

### Metabolic disease initiates due to the self-reinforcing properties of tissue RAS

**Figure 1:**
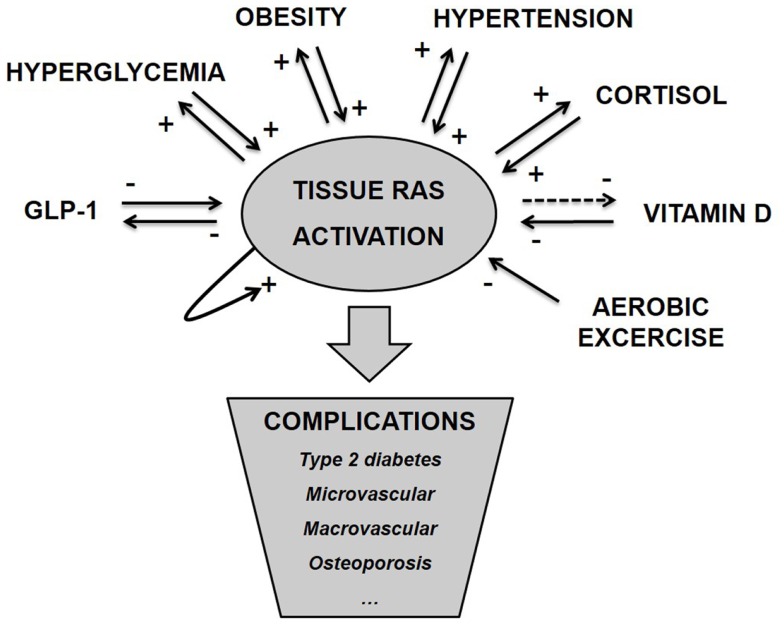
**Tissue RAS activity is controlled by several well-known metabolic hormones and conditions**. This control often involves positive feedback mechanisms, which makes the basis for an unstable system and progression of disease. We hypothesize that metabolic complications arise as a consequence of pathological activated tissue RAS.

As argued above and illustrated in Figure 1, tissue RAS is regulated by well-known risk factors of diabetes and through several positive feedback mechanisms. ANG II even seems to stimulate its own secretion (71). This makes the basis for an unstable system and we hypothesize that T2D and other metabolic diseases develop when tissue RAS becomes self-reinforcing. This may happen due to one far-off parameter (hyperglycemia in type 1 diabetes (T1D) or high dose glucocorticoid treatment) but is often caused by a sum of risk factors, like the combination: obesity, inactivity, high glycemic index diet, and VitD deficiency.

### Metabolic complications are caused by increased tissue RAS activity

The multiple different effects of ANG II depend on which tissue/cell is involved, the receptor, and the time frame of stimulation. Many of ANG II’s immediate effects (e.g., vasoconstriction) are mediated via the AT_1_R through classical G-protein-dependent signaling pathways (72).

Stimulation of the AT_1_R also activates the membrane-bound NAD(P)H oxidase leading to increased generation of reactive oxygen species (ROS). The NAD(P)H formed ROS may activate mitochondrial K_ATP_ channels to additionally burst mitochondrial ROS generation (73). Alternatively, the mitochondrial ROS formation may be mediated via local mitochondrial ANG II sensitive receptors (74). After its generation, intracellular ROS can activate many down-stream molecules such as mitogen-activated protein kinases, protein tyrosine phosphatases, protein tyrosine kinases, and transcriptional factors, which if chronically activated will promote inflammation, atherosclerosis, thrombosis, and fibrogenesis (72, 73).

In almost all organ systems, RAS activation has been associated with degeneration, tissue remodeling, and dysfunction that are likely secondary to ROS formation and phenotypic known as the metabolic syndrome. Practically all complications in diabetes are related to the RAS and increased ANG II levels including ischemia, myocardial infarction, stroke, nephropathy, cardiomyopathy, retinopathy (75), polyneuropathy (76), and erectile dysfunction (77).

We therefore hypothesize that the majority of complications seen in metabolic disease are consequences of a pathological-activated tissue RAS. Thus, hyperglycemia may not directly induce complications apart from the ability to stimulate tissue RAS. Although this statement may sound controversial, we do not find it to conflict with the leading theory on the pathogenesis of diabetic complications, The Brownlee hypothesis (78). The Brownlee hypothesis states that all pathological metabolic pathways induced by hyperglycemia are secondary to increased ROS (78). We just add an extra link to the chain so that hyperglycemia stimulates ROS production via stimulation of tissue RAS. By introducing ROS formation secondary to tissue RAS activation as a unifying mediator of complications, we have plausibly linked all the major risk factors in metabolic disease.

Essentially, the full theory simplifies T2D to a complication of a malfunctioning tissue RAS which places it next to other complications. However, the “T2D complication” may be special in the ability to accelerate RAS over-activity (hyperglycemia induced) and thereby other complications. According to the hypothesis, tissue RAS must be pathologically activated prior to the onset of T2D and this may explain why complications often exist at the time of T2D diagnosis. By contrast, the complications seen in T1D will only occur if glucose level is uncontrolled and allowed to secondarily activate tissue RAS.

Both T1D and T2D are associated with a markedly increased risk of bone fractures despite increased bone mineral density in obese T2D patients (79, 80). Tissue RAS is also present in bones and involved in bone remodeling (81). In an eight-week interventional study, ARBs improved bone mass and strength in osteoporotic rat femurs (82). Consistent with this, an observational study demonstrated that ARB treatment significantly reduced fracture risk (hazard ratio 0.76) in older adults (83). We therefore hypothesize that the osteoporotic complication is caused primarily by an activated bone tissue RAS and that the positive VitD effects on bone metabolism are partly due to RAS-inhibition. Equally, the well-known pro-osteoporotic properties of cortisol as well as the less-known anti-osteoporotic properties of GLP-1 (84, 85) are caused by bone tissue RAS stimulation and inhibition respectively.

## Discussion

Tissue RAS is regulated by multiple factors of which we have only mentioned some. We have focused on the interaction between tissue RAS and the major metabolic hormones and conditions, but this is certainly not the complete story. Tissue RAS is present in almost every tissue and exert multiple different actions determined by the involved cells. Thus, we speculate that tissue RAS should be seen as a common effectuating machinery required for multiple different purposes.

From an evolutionary point of view, it is cost-effective to have a common system to potentiate and effect the actions of the regulating hormones. Interestingly, the RAS is also found in primitive animals without a closed circulatory system (86), which indicate that the system is far more than a mediator of vasoconstriction.

The RAS offers multiple targets for regulation as we have seen (AGT, renin, AT_1_R, ACE, down-stream signaling) and several more which we have excluded (prorenin receptor, ACE2, AT_2_R, AT_4_R, neutral endopeptidase, chymase, Mas receptor). We have focused on the ANG II–AT_1_R interactions since these are the best described and considered the most important. However, the system is complex and several other components probably play significant roles as well. Especially, the AT_2_Rs have come in focus in recent years and seem to be important counter-regulators of AT_1_R function (87). This may be of particular importance when the AT_1_Rs are pharmacologically blocked.

While we have focused only on a few regulators of tissue RAS the same is true regarding the implications of the hypothesis. We will likely identify that many known disorders are caused by a malfunctioning tissue RAS in specific organs and these could likely include inflammatory bowel disease, psoriasis, preeclampsia, Alzheimer’s disease, and depression.

It is essential to emphasize the importance of the local nature of tissue RAS and it is somewhat misleading to talk about a general over-activity. Even the circulating classical RAS should to some degree be considered a local independent system when compared to the tissue level systems. However, since tissue RAS in different tissues have many regulatory hormones and conditions in common, it makes sense to talk about a general direction of activity level. Additionally, although paracrine acting, there seem to be a degree of “spill over” of RAS components from one tissue to another. This is of course very pronounced with the classical circulating RAS but adipocyte AGT synthesis, as an example, also contributes significantly to the total AGT pool.

The local environments with respect to angiotensin concentrations and angiotensin sensitivity are generally hard to assess, especially in humans. In addition, evidence suggests that besides paracrine and autocrine actions, ANG II exerts intracrine effects mediated by intracellular located receptors. ANG II may be internalized or act immediately after intracellular synthesis without ever leaving the cell (2, 88). This may partly explain why the importance of tissue RAS is still broadly unacknowledged and why classical extracellular RAS inhibitors in many ways are insufficient.

If the phenotypes of metabolic diseases and the complications seen in relation hereto are caused by locally activated tissue RAS as hypothesized, diagnosing and treating an activated RAS become the primary target in the clinic. Screening for activated tissue RAS in relevant tissues instead of elevated glycemic level would theoretically serve as an early and more efficient marker of the risk of complications – including T2D. The urinary excretion of AGT has been proposed as a non-invasive marker of renal tissue RAS activity (89). Although this particular method may be imperfect (90), the concept of assessing specific tissue RAS activity may be of great diagnostic value in the future.

Provided that the proposed hypothesis is correct, it should theoretically be possible to prevent T2D by securing that tissue RAS never reaches a high or self-reinforcing level, which emphasizes the importance of prophylaxis and early intervention. Besides lifestyle interventions, drugs aiming to decrease tissue RAS should be preferred and drugs targeting the intracellular part of tissue RAS or some of the newer extracellular components may be the drugs of tomorrow.

A few studies suggest that the ANG II-induced ROS formation is mediated through AT_1_Rs located directly on the mitochondrial membranes (74), whereas one study cannot confirm this relationship (91). If indeed existing and functional, these mitochondrial AT_1_Rs seem particular attractive to block selectively to prevent complications.

The hypothesis obviously needs further investigation. While several interactions with tissue RAS are well described, their individual importance is less characterized. In fact, the importance of tissue RAS, in general, is poorly understood and according to this hypothesis greatly underestimated. The local nature of the systems, however, makes the design of appropriate experiments harder. Interesting pathways can be elucidated *in vitro* but the importance *in vivo* can be difficult to extrapolate. One possible experimental approach is the use of AT_1_R knock-out mice. According to the hypothesis, these mice would have defective responses to hormones, which primarily depend on tissue RAS.

In conclusion, we have hypothesized that the tissue RAS is in the very center of metabolic regulation and is the true effector of multiple hormones. Implications of this hypothesis suggest a new unifying understanding of the nature, initiation, and complications of metabolic disease.

## Conflict of Interest Statement

Jørgen Frøkiær has no conflicts of interests. Jeppe Skov is employed by Novo Nordisk in a PhD fellowship. Frederik Persson owns stock in Novo Nordisk has received research grants from Novartis, and speaker honoraria from Novartis, Eli Lilly, and Boehringer Ingelheim. Jens Sandahl Christiansen is a recipient of unrestricted research grants from Novo Nordisk, a clinical investigator for Novo Nordisk, receives lecture fees from Novo Nordisk, Eli Lilly, and Pfizer and is a member of Novo Nordisk advisory boards.
